# Anatomic Repair of Congenitally Corrected Transposition: Reappraisal of Eligibility Criteria

**DOI:** 10.1007/s00246-022-02841-z

**Published:** 2022-02-11

**Authors:** Viktoria H. M. Weixler, Peter Kramer, Peter Murin, Olga Romanchenko, Mi-Young Cho, Stanislav Ovroutski, Michael Hübler, Felix Berger, Joachim Photiadis

**Affiliations:** 1 Department of Congenital Heart Surgery, German Heart Center Berlin, Augustenburger Platz 1, 13353 Berlin, Germany; 2 Department of Congenital Heart Disease/Pediatric Cardiology, German Heart Center Berlin, Augustenburger Platz 1, 13353 Berlin, Germany

**Keywords:** Congenital heart disease, Congenital heart surgery, Great vessel anomaly, Ventricular septal defect, Arterial switch operation, Echocardiography

## Abstract

Several criteria to identify suitable candidates for anatomic repair in congenitally corrected transposition (cc-TGA) have been proposed. The purpose of this study was to critically re-evaluate adequacy of these recommendations in our patient cohort. All cc-TGA patients undergoing anatomic repair between 2010 and 2019 were reviewed. Evaluated eligibility criteria for repair included age ≤ 15 years, LV mass index ≥ 45–50 g/m^2^, LV mass/volume ratio > 0.9–1.5 and systolic LV to right ventricle pressure ratio > 70–90% among others. Repair failure was defined as postoperative early mortality or LV dysfunction requiring mechanical circulatory support. Twenty-five patients were included (median [interquartile range] age at surgery 1.8 years [0.7;6.6]; median postoperative follow-up 3.2 years [0.7;6.3]). Median preoperative LV ejection fraction was 60% [56;64], indexed LV mass 48.5 g/m^2^ [43.7;58.1] and LV mass/volume ratio 1.5 [1.1;1.6], respectively. A total of 12 patients (48%) did not meet at least one of the previously recommended criteria, however, all but two patients (92%) experienced favorable early outcome. Of 7 patients (28%) with indexed LV mass < 45 g/m^2^, 6 were successfully operated. There were two early repair failures (8%) with LV dysfunction: one patient died and one required mechanical circulatory support but recovered well. Surgery was performed successfully in patients with LV mass and volume *Z*-scores as low as − 2 and − 2.5, respectively. Anatomic correction for cc-TGA can be performed with excellent early outcome and is feasible even in patients with LV mass below previously recommended cut-offs. The use of LV mass and volume *Z*-scores might help to refine eligibility criteria.

**Supplementary Information:**

The online version contains supplementary material available at 10.1007/s00246-022-02841-z.

## Introduction

Congenitally corrected transposition of the great arteries (cc-TGA) is a rare and complex congenital malformation, accounting for approximately 0.05% of all congenital heart defects [1]. Untreated, natural history is often accompanied by progressive failure of the systemic right ventricle (RV), tricuspid valve (TV) regurgitation, arrhythmia as well as sudden cardiac death with approximately 50% of the patients dying before the age of 40 years [1, 2].

Different types of anatomic repair aiming for correction of the double discordance have been proposed, combining an atrial stage switch (Senning or Mustard procedure) together with either arterial switch operation (ASO) as double switch operation or, in case of relevant left ventricular outflow tract obstruction (LVOTO) and ventricular septal defect (VSD), as intra-ventricular rerouting by means of a Rastelli procedure [3]. More recently, variations of aortic root translocations such as the Nikaidoh or the half-turned truncal switch/*en bloc* rotation procedures have been introduced, improving LVOT geometry and supposedly avoiding the concern of recurrent LVOTO [4–6].

Promising early outcomes after anatomic correction have been reported [7–10], however, there is uncertainty regarding the crucial prerequisites that have to be met in order to ensure that the subpulmonary morphologic left ventricle (LV) will sustain the workload of the systemic circulation after surgery. Several studies have proposed various selection criteria for the evaluation of cc-TGA patients’ eligibility for anatomic repair. Suggested criteria mostly focus on LV pressure load and muscle mass, however their appropriateness has not been broadly validated [11–13]. Moreover, it remains unclear, to what extent such criteria apply to cc-TGA patients with an unrestrictive VSD. Although in general these patients might be perceived as suitable for anatomical correction since the morphological LV is supposedly “well-trained” due to continuous systemic pressure load and volume load, it is not self-evident that this will naturally translate into sufficient ventricular size or muscle mass development [14].

The purpose of this study was to re-evaluate previously recommended criteria for cc-TGA in our institutional cohort in respect to their adequacy in identifying patients suitable for anatomic correction.

## Patients and Methods

### Study Design

We performed a retrospective analysis of all cc-TGA patients that underwent anatomic repair from 01/2010 to 12/2019 in our institution. No physiologic repair was performed at our institution during the study period. The study was approved by the institutional ethics committee (EA2/165/19), the requirement of written consent was waived.

### Surgical Procedures

**Fig. 1:**
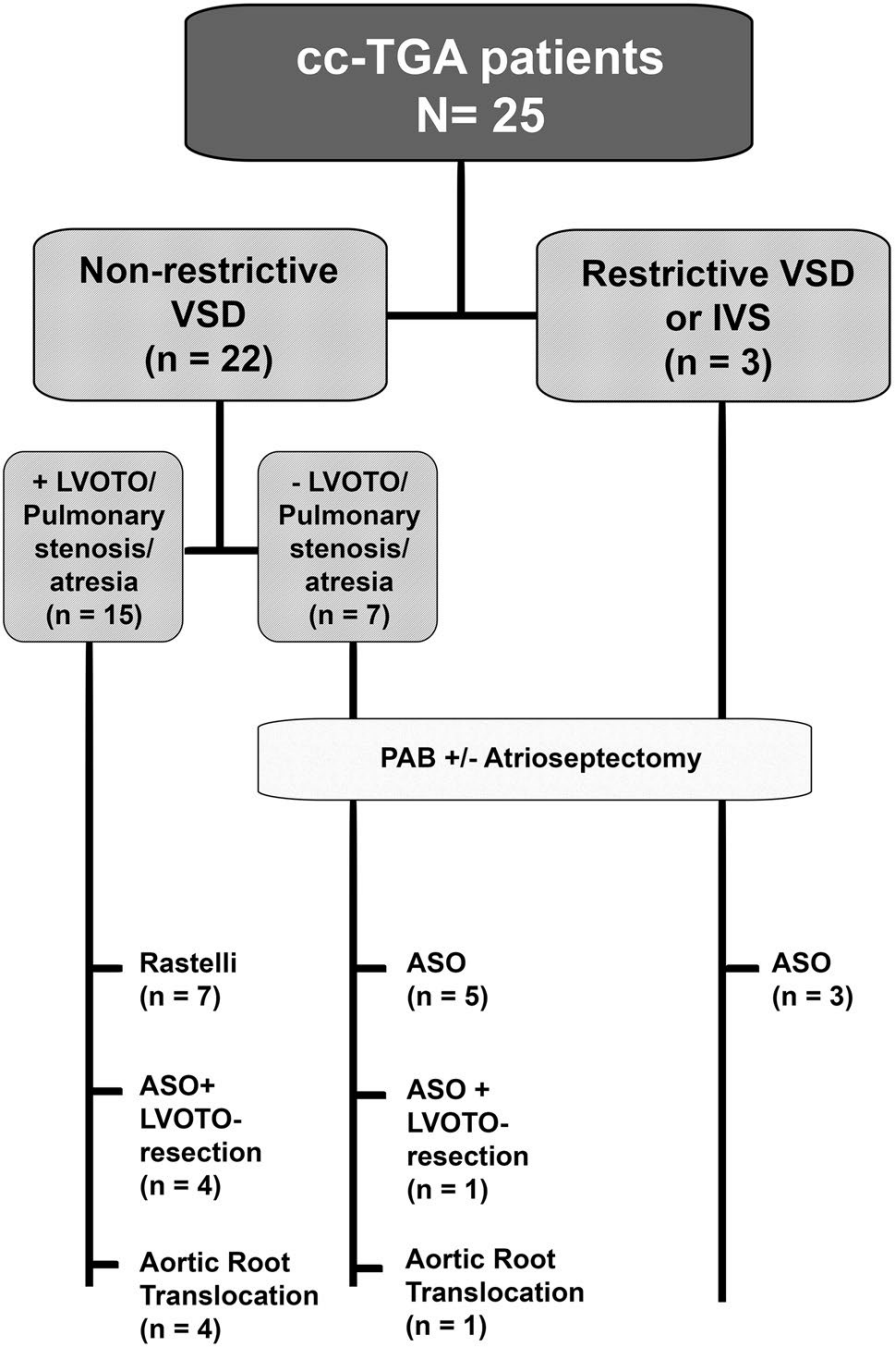
Description of sthe study cohort. *ASO* arterial switch operation, *cc-TGA* congenitally corrected transposition of the great arteries, *IVS* intact ventricular septum/ no ventricular septal defect, *LVOTO* left ventricular outflow tract obstruction, *PAB* pulmonary artery banding, *VSD* ventricular septal defect

Various procedures representing anatomic repair have been employed in our institution as deemed technically feasible and promising favorable surgical results according to individual cardiac morphology (Fig. 1). Correction of atrio-ventricular discordance by atrial stage switch was performed as Senning or Mustard procedure. Correction of ventriculo-arterial discordance was carried out as ASO, solely or in combination with LVOTO resection, Rastelli procedure or aortic root translocation, either performed as classical Bex-Nikaidoh procedure or as *en bloc* rotation (half-turned truncal switch), respectively [4–6, 14]. In patients with significant LVOTO, aortic root translocation was the preferred surgical approach, if feasible according to coronary anatomy and pulmonary valve function and dimension. Patients with non-existing/restrictive ventricular septal defect (VSD) underwent initial pulmonary artery banding (PAB) to train the morphological LV by increasing afterload. Additionally, an atrial septal defect was created in cases without preexisting atrial communication during the more recent study period to increase LV preload. In patients with a large VSD and non-existing/insufficient LVOTO, PAB was established to protect the pulmonary vasculature until correction. A systemic-to-pulmonary shunt was placed in patients with severe restriction of pulmonary blood flow and desaturation prior to surgical correction.

### Criteria for Anatomic Repair and Outcome Definitions

The following previously recommended criteria to identify suitable candidates for anatomic repair were evaluated in our patient cohort: age ≤ 15 years, LV ejection fraction (EF) > 55%, mitral valve (MV) regurgitation ≤ mild, LV mass index ≥ 45–50 g/m^2^, LV mass/volume ratio > 0.9–1.5 and systolic LV/RV pressure ratio > 70–90%, [8, 11–13, 15, 16].

Data pertaining to these criteria were retrieved from medical charts. Additional data recorded and analyzed included preoperative demographics, functional status, morphological details as well as prior palliative surgical procedures; intraoperative data as well as early postoperative complications were also extracted. Primary endpoint was early repair failure which was defined as early mortality (30-day mortality) and/or postoperative LV failure requiring mechanical circulatory support. Secondary endpoints were late repair failure defined as severe impairment of LV function and/or heart failure corresponding to NYHA/modified Ross classification [17] functional class ≥ III at last follow-up; additional secondary endpoints included mid-term mortality, requirement of reoperation and/or catheter reintervention.

### Echocardiographic Analysis

Patients’ digitally archived echocardiograms at baseline and last follow-up were analyzed offline with a vendor-specific software (EchoPAC™ version 203; General Electric Vingmed Ultrasound AS, Horten, Norway). Measurements were performed according to current recommendations [18]. Atrioventricular or semilunar valve regurgitation was graded semi-quantitatively as absent/trace, mild, moderate or severe according to color Doppler jet area and vena contracta width. Speckle tracking analysis with measurement of RV and LV global systolic strain was performed in standard apical four-chamber view greyscale images [19, 20]. *Z*-scores for echocardiographic measurements were calculated according to published regression equations [19, 21–23]. *Z*-scores for RV fractional area change were estimated from published mean values and standard deviations [24].

### Statistical Analysis

Variables are presented as median [interquartile range] for continuous variables or as frequency (%) for categorical data. Comparisons between unpaired groups were performed by nonparametric Mann–Whitney *U* test or one-way ANOVA with Bonferroni post-hoc test according to number of groups compared. Paired variables were compared using Wilcoxon signed rank test. Freedom from death and reoperations were analyzed using Kaplan–Meier time-to-event models and log-rank test was used to compare groups. Multivariable binary logistic regression analysis was performed to evaluate independent risk factors for primary outcome. Statistical analysis was performed using IBM-SPSS version 24.0 (IBM-SPSS Inc, Armonk, NY) and GraphPad Prism version 8.4.0 (GraphPad Software, Inc., San Diego, CA, USA). *P*-values < 0.05 were considered statistically significant.

## Results

### Baseline Characteristics

A total of 25 patients underwent anatomic correction in our institution during the study period. Median follow-up was 3.2 years [0.7;6.3]. Patient’s characteristics are detailed in Table 1. Eighteen patients (72%) had undergone prior surgical procedures. PAB was performed at a median age of 2.8 months [1.4;34.9] and median time interval between PAB and anatomic correction was 9.9 months [4.9;10.1]. Preoperatively, heart failure of varying extent was common; most patients were in NYHA/modified Ross classification functional class II (*n* = 16, 64%), seven (28%) were in class III and only two patients (8%) were asymptomatic (class I).

**Table 1:** Baseline patients’ characteristics

Variable	Patients (*N* = 25)
Male gender	16 (64%)
Body weight (kg)	10.2 [7.5;20.5]
Age at surgery (years)	1.8 [0.7;6.6]
Associated lesions	
Non-restrictive VSD	21 (84%)
LVOTO	11 (44%)
Pulmonary stenosis/atresia	12 (48%)
LVOTO ± pulmonary stenosis/atresia	15 (60%)
Coronary anatomy	
1R2LCx	14 (56%)
1RLCx	4 (16%)
1L2CxR	3 (12%)
1LCx2R	3 (12%)
1RL2Cx	1 (4%)
Segmental notation	
{SLL}	20 (80%)
{IDD}	5 (20%)
Anatomic variations	
Dextrocardia	7 (28%)
Persistent left superior vena cava	2 (8%)
TAPVD/PAPVD	2 (8%)
Right-sided aortic arch	2 (8%)
Interrupted inferior vena cava	1 (4%)
Prior palliative procedures	
Systemic-to-pulmonary shunt	11 (44%)
PAB	10 (40%)
Glenn procedure	4 (16%)
VSD closure	2 (8%)
Atrioseptostomy	1 (4%)
Damus-Kaye-Stansel procedure	1 (4%)

Continuous data presented as median [interquartile range]; categorical data are presented as frequency (%)

*LVOTO* left ventricular outflow tract obstruction, *PAB* pulmonary artery banding; *TAPVD/PAPVD* total/partial anomalous pulmonary venous drainage, *VSD* ventricular septal defect. Several patients had more than one prior surgical procedure

### Preoperative Invasive and Echocardiographic Evaluation

All patients had systemic or near systemic LV pressure load. A non-restrictive VSD was present at the time of anatomic repair in 21 patients (84%). Four patients (16%) had mildly reduced LVEF < 55% with the lowest preoperative LVEF being 50%. Of note, nine patients (36%) had abnormally low LVEDV *Z*-scores <  − 2. Indexed LV mass was below 45 g/m^2^ in seven patients (28%), however, LV mass *Z*-score was marginally below − 2 in only one patient, while the remaining patients had *Z*-scores > − 2. Only one patient showed more than mild MV regurgitation. RV function was also generally sufficiently preserved. Moderate or severe TV regurgitation was documented in six (24%) patients (Table 2, Supplemental Table 1).

**Table 2:** Echocardiographic and hemodynamic parameters

Variable	Preoperatively (*N* = 25)	Last follow-up (*N* = 22)
RV echocardiographic measurements		
RV diastolic area (cm^2^)	8.4 [5.2;13.7]	9.0 [5.9;10.5]
RV diastolic area *Z*-score	0.3 [− 0.3;1.5]	− 0.1 [− 1;0.4]
RVOT pressure gradient (mmHg)	5.0 [5.0;7.5]	22.0 [11.8;38.0]
RV fractional area change (%)	35.7 [30.6;47.2]	43.7 [35.9;47.5]
RV fractional area change *Z*-score	− 0.5 [− 0.8;0.8]	0.8 [− 0.2;1.5]
RV global systolic strain	− 16.3 [− 11.3; − 17.7]	− 17.5 [− 15.6; − 19.7]
TAPSE (mm)	10 [9.0;12.5]	11.5 [9.0;13.3]
TAPSE *Z*-Score	− 3.8 [− 5.5; − 2.5]	− 4.8 [− 6.4; − 2.9]
Pulmonary valve diameter *Z*-score	− 1.7 [− 3.4; − 0.37]	
LV echocardiographic measurements		
LVEDVi (ml/m^2^)	36.9 [29.9;43.9]	48.4 [41.2;61.7]
LVEDV *Z*-score	− 1.7 [− 2.1; − 1.2]	− 0.4 [− 1.2; − 0.1]
LVOT pressure gradient (mmHg)	65 [55;80]	6 [4;10]
LV mass index (g/m^2^)	48.5 [43.7;58.1]	61.3 [56.3;71.3]
LV mass *Z*-score	0.5 [− 0.3;1.9]	1.1 [0.2;3.4]
LV mass/volume ratio	1.5 [1.1;1.6]	1.2 [0.9;1.4]
LV EF (%)	60 [56;64]	56 [49;61]
LV global systolic strain (%)	− 21.2 [− 17.3; − 24.2]	− 17.9 [− 15; − 21.1]
LV global systolic strain *Z*-score	0.4 [− 1.9;1.6]	− 1.4 [− 3.1;0.9]
Aortic valve diameter *Z*-score	3.0 [2.3;4.6]	2.8 [0.9;4.4]
Invasive measurements		
LV end-diastolic pressure (mmHg)	10 [8;12]	
Systolic LV/RV pressure ratio	1.00 [0.99;1.02]	

Data presented as median [interquartile range]

*EF* ejection fraction, *LV* left ventricle, *LVEDV* left ventricular end-diastolic volume, *LVEDVi* indexed left ventricular end-diastolic volume, *LVOT* left ventricular outflow tract, *RV* right ventricle, *RVOT* right ventricular outflow tract, *TAPSE* tricuspid annular plane systolic excursion

### Surgical Procedures and Postoperative Complications

Various surgical procedures were performed as depicted in Fig. 1. Atrial switch was predominantly carried out as Senning procedure (*n* = 23, 92%) while one patient received a Mustard procedure and another a hemi-Mustard procedure in conjunction with a bidirectional Glenn anastomosis. A Lecompte maneuver was performed in five patients (20%) with anterior–posterior relationship of the great arteries. Two patients underwent TV repair and one patient MV repair during anatomic repair. Intraoperative and early postoperative variables are given in Supplemental Table 2. Several patients experienced postoperative complications with dysrhythmias being the most frequent. Three patients (12%) required pacemaker implantation. No significant differences were noticed in intraoperative/early postoperative characteristics between different surgical techniques or between patients with or without prior PAB (all *P* > 0.05).

### Early Repair Failure

Early repair failure occurred in two patients (8%). The first patient died, corresponding to an early mortality rate of 4%, while the second patient suffered from early postoperative LV dysfunction (Supplemental Table 1). The deceased patient (#10) with a large VSD, relevant LVOTO and significant biventricular hypertrophy was almost 18 years at the time of anatomic correction with no prior palliative procedure. She underwent Senning and aortic root translocation and postoperatively required mechanical circulatory support due to biventricular systolic failure. Unfortunately, she could not be weaned off support and died from multi-organ failure.

The second patient (#22) underwent PAB shortly after birth due to a large VSD with unrestricted pulmonary blood flow. With a LV mass index of 35.8 g/m^2^ (*Z*-Score − 1.3) he underwent Senning with ASO but could not be weaned from bypass due to severe LV dysfunction. He required mechanical circulatory support in form of extracorporeal membrane oxygenation which was later switched to a ventricular assist device. Myocardial function, however, recovered and the patient could successfully be weaned 3 months postoperatively.

### Mid-Term Outcomes

No late repair failure occurred. Nonetheless, late mortality was 8.3% (2/24) due to re-operation mortality. One patient (#7) developed severe pulmonary baffle stenosis one year after anatomic correction and died the day before scheduled urgent re-operation from refractory cardiac arrest. The second patient (#4) showed severe pulmonary heterograft stenosis and additional mild pulmonary venous baffle stenosis requiring complex reoperation 2 years after anatomic repair. He could not be weaned from bypass and required circulatory support. Unfortunately, myocardial function did not recover.

Reoperation rate was 16.6% (4/24) during mid-term follow-up, including pulmonary venous baffle stenosis repair in two (8.3%) and pulmonary valve heterograft replacement in two (8.3%) patients, in one of whom additional baffle stenosis repair was performed. One patient (4.2%) underwent resection of re-occurring LVOTO after an initial ASO and LVOTO resection. Four patients (16.6%) required catheter reinterventions with balloon dilatation of the pulmonary arteries. No significant differences regarding frequency of reoperations or re-interventions were found between the different surgical techniques or between patients with or without prior PAB (all *P* > 0.05).

**Fig. 2:**
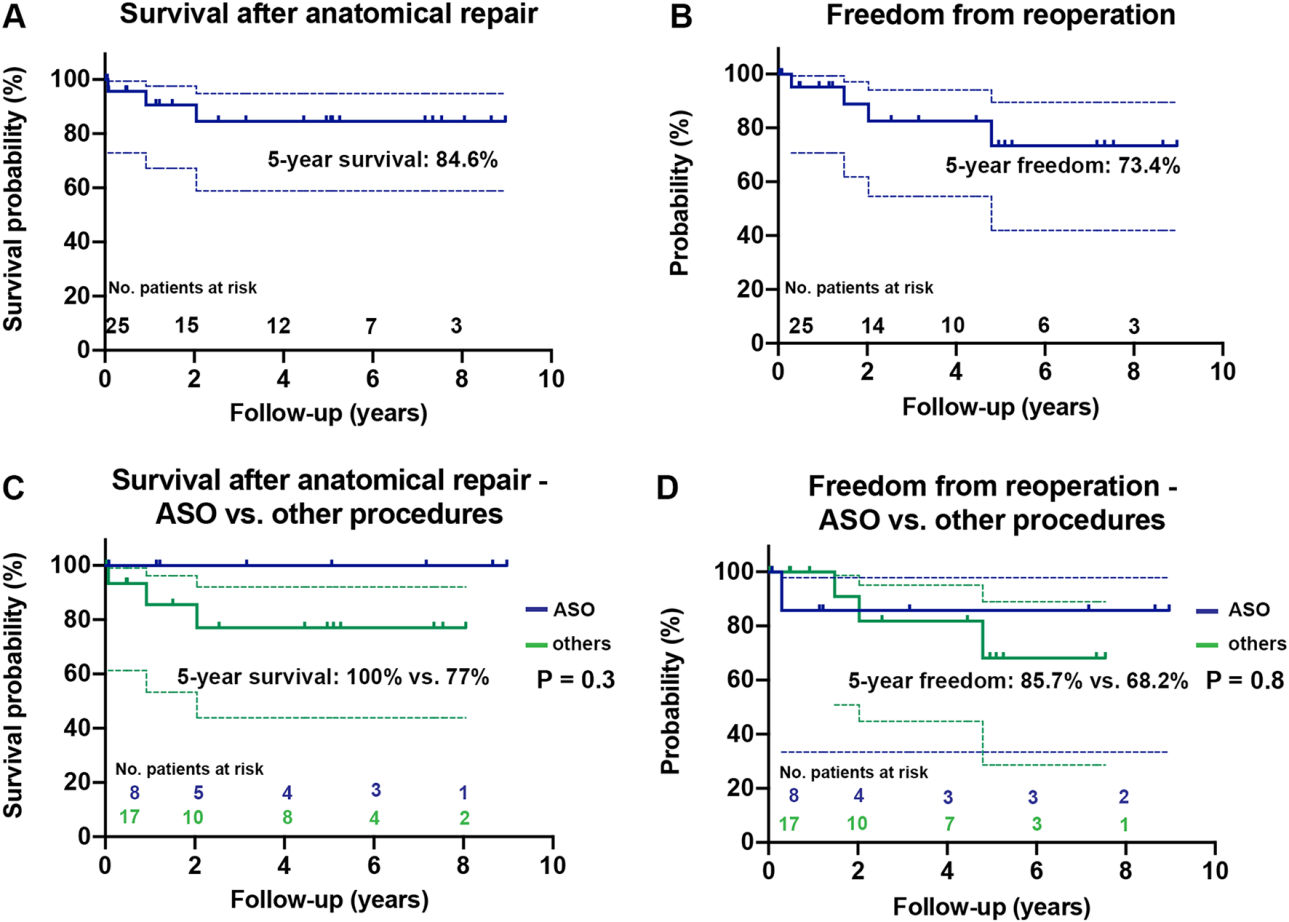
Survival and freedom from reoperation estimates. Kaplan–Meier curves (solid lines) with 95%-confidence intervals (dashed lines) depicting survival and freedom from reoperation in entire study cohort (**a**, **b**) and anatomical repair with atrial stage switch and arterial switch operation (ASO) versus other procedures (**c**, **d**). The color image is available in the online version of the article

5-year survival estimate was 84.6% [95% CI 59.9;94.9] and 5-year freedom from reoperation rate was 73.4% [95% CI 42.0;89.6] in the entire study population. In patients with ASO, 5-year survival rates and 5-year freedom from re-operation did not differ significantly compared to the more complex procedures [100% vs. 77% (95% CI 43.9;92.1), *P* = 0.3 and 85.7% (95% CI 33.4;99.8) vs. 68.2% (95% CI 28.6;88.9), *P* = 0.8, respectively. Figure 2]. Also, comparing patients with and without prior PAB, there was no significant difference concerning 5-year survival and freedom from reoperation rates [100% vs. 75.9 (95% CI 42.23;91.6), *P* = 0.2 and 70% (95% CI 22.5;91.8) vs. 72% (95% CI 23.8;92.8), *P* = 0.5, respectively].

At last follow-up, all surviving patients were in NYHA/modified Ross classification functional class I (15/22, 68%) or II (7/22, 32%) and there was no case of LV failure occurring during follow-up.

### Echocardiographic Follow-Up Data

The majority of patients exhibited good LV function and preserved LV global systolic strain at last follow-up although in general, LVEF decreased slightly but statistically significantly (*P* = 0.005, Table 2). Seven patients (32%) had mild (LVEF < 55%) and one patient (5%) moderate impairment of LV function (LVEF < 45%) which was, however, stable during follow-up. Overall, LVEDV *Z*-scores increased significantly (*P* = 0.005, Table 2). Aortic/neoaortic valve regurgitation was either absent/trivial (13/22, 59%) or mild (9/22, 41%). Also, MV incompetence was uncommon with only three patients (14%) having mild regurgitation. Moreover, TV was competent in most patients (77.3%) while only five (22.7%) had mild regurgitation. No differences in any of these parameters were noticed between different surgical techniques (all *P* > 0.5).

### Risk Factors for Early Failure After Anatomic Repair

A multivariable regression analysis to identify potential risk factors (age, surgical method, prior PAB, relevant LVOTO, low LV mass) for early repair failure revealed no independent risk factors (*P* = 0.2–0.9, Supplemental Table 3).

**Table 3:** Proposed eligibility criteria for cc-TGA patients to undergo anatomic repair

Parameter	Value
LV EF%	≥ 50%
LV mass *Z*-Score	≥ − 2; < 3
LVEDV *Z*-Score	≥ − 2.5
LV mass/volume ratio	≥ 0.9; ≤ 1.8
LV/RV pressure ratio	≥ 0.9

*EF* ejection fraction, *LV* left ventricle, *LVEDV* left ventricular end-diastolic volume, *RV* right ventricle

## Discussion

Patients with cc-TGA constitute a complex and heterogeneous cohort frequently associated with cardiac anomalies, anatomic variations and individual hemodynamic characteristics rendering the anatomic correction technically challenging. In our study, we demonstrate that excellent early and mid-term survival rates are achievable. This is consistent with previous studies reporting 5-year survival rates varying between 75 and 95% [7, 9, 10].

To achieve favorable outcome after anatomic correction, careful and accurate selection of appropriate patients, optimal timing and surgical technique are clearly important. Several studies have proposed various criteria to assess eligibility of cc-TGA patients to undergo anatomic repair, mainly focusing on patient’s age and morphologic LV function, pressure, mass and mass/volume ratio [8, 11–13, 15].

Age as factor influencing outcome and survival has been the focus of various studies, generally in respect of age limit for a meaningful re-training of the morphological LV, which has been reported to be of limited success beyond the age of 15 years [8, 15]. It is however unclear, if such age limit is an appropriate selection criterion for anatomical correction if systemic pressure load of the morphological LV is already present. In our patient cohort, 2 patients were beyond this age limit at time of surgery. One patient (#4) initially experienced favorable outcome after anatomical repair but unfortunately died 2 years later after reoperation for pulmonary heterograft stenosis. The second patient (#10) had early repair failure. She displayed severely compromised biventricular function postoperatively, probably due to considerable myocardial hypertrophy not tolerating cardiopulmonary bypass. Our results suggest that age > 15 years is not a contraindication for anatomic correction per se if LV volume and pressure lead are already present, but also emphasize the importance of careful patient selection in respect to additional criteria.

LV function and MV competence represent additional important factors to consider when evaluating a cc-TGA patient for anatomic correction. A normal LVEF (> 55%) and MV regurgitation less than mild/moderate have been proposed as required by several groups [11, 12]. In our cohort, four patients with favorable early outcome had mildly impaired LVEF (50–53%) indicating that minor functional impairment does not represent a contraindication.

Several studies have focused on morphologic LV pressure in relation to systemic pressure as major criterion to determine suitability for anatomic correction and LV/RV pressure ratios ranging from > 70 to ≥ 90% have been recommended [8, 11, 12]. All our patients had LV/RV pressure ratios of ≥ 90% and therefore our data does not substantiate conclusions regarding the suitability of lower LV pressure loads.

The most disputable criterion might be the suggested LV mass index required to be at least 45–50 g/m^2^ [11]. In fact, seven of our patients with successful anatomic corrections (28%) had values below 45 g/m^2^ as determined by echocardiography. LV mass has a strong relationship to body surface area, however, the relation is non-linear and accordingly, reference values for echocardiographically determined LV mass index are not constant [21]. It would thus seem advisable to establish thresholds in relation to normal values accounting for growth-dependent changes using *Z*-scores. LV mass *Z*-Scores of these patients were normal in six (− 1.3 to − 0.1) and borderline abnormal in one patient (− 2.1), respectively. The two patients with early repair failure both had normal or above normal LV mass *Z*-scores (− 1.3 patient #22 and 4.1 patient #10, respectively), emphasizing that LV mass alone seems inadequate to determine suitability. Moreover, significant LV hypertrophy as observed in one of these patients (#10), might represent a risk factor for surgical failure hitherto not sufficiently taken into consideration.

Furthermore, small LV dimensions may constitute a possible risk factor for early LV failure not adequately appreciated in previous recommendations. While previous studies focused on sufficient LV mass in relationship to volume suggesting a lower limit for mass/volume ratio of 0.9–1.5 [12, 16], the possible negative impact of postoperative LV systolic but also diastolic dysfunction which might be caused by small LV dimensions and/or hypertrophy are not reflected by this threshold [12]. It might be reasonable to additionally implement upper thresholds for LV mass/volume ratio as well as LV mass in the considerations when evaluating patients for anatomic repair. In our cohort, all patients met the previously suggested criterion of mass/volume ratio > 0.9. Patients however generally tended to have LV volumes in the lower range of the normal spectrum with a median LVEDV *Z*-score of − 1.7 [− 2.1; − 1.2] despite most patients having an unrestrictive VSD with systemic pressure load and volume load. Nine patients (36%) had abnormally low *Z*-scores <  − 2 prior to surgery. The patient (#22) who experienced early postoperative LV failure had the lowest LVEDV *Z*-score in our cohort with − 2.93. Retrospectively, we would judge that this patient most likely would have required longer LV training with possibly additional volume load by the creation of an interatrial communication to enhance LV volume load. Moreover, from out data we conclude that it may not generally be assumed that presence of a large unrestrictive VSD with LV pressure and volume load renders cc-TGA patients suitable candidates for repair in respect to sufficient LV muscle mass and volume development. Rather, these patients with cc-TGA and unrestrictive VSD likewise require a comprehensive and careful evaluation of their eligibility for anatomic repair.

As important finding of this study, a substantial number of our patients did not meet previously recommended criteria for “ideal” candidates for anatomic correction but nevertheless underwent successful anatomic repair. Out of 23 patients successfully operated, 10 (44%) would have been classified as “unsuitable” candidates if these criteria were applied strictly [8, 11–13, 15, 16]. Summarizing our data, we propose modified criteria to select patients suitable for anatomic correction which are tabulated in Table 3.

### Limitations

Our study has several important limitations. Since this is a retrospective, single institutional non-interventional study, patients were managed individually and moreover, evaluation and treatment protocols changed during the study period. Echocardiographic assessment of important parameters regarding ventricular function, dimensions and mass has some significant technical limitations. Suboptimal image acquisition as well as abnormal ventricular geometry may represent considerable sources of error regarding measurements. As additional important note, reference values and corresponding *Z*-scores for pediatric cardiac structures vary substantially according to measuring methods and imaging modalities and are generally derived from normal anatomies. Future studies should preferably employ standardized imaging protocols considering these issues. Moreover, it might be recommendable to favor more accurate and less variable imaging modalities such as cardiac magnetic resonance imaging to assess suitability for anatomic correction, which was, however, only inconsistently performed in our study and therefore not analyzed.

In addition, our small cohort size and short follow-up interval clearly limit the possibilities of statistical analyses.

## Conclusions

Our study demonstrates that anatomic correction for cc-TGA patients can be performed with good early and mid-term outcomes but also indicates that previously recommended eligibility criteria may require reconsideration. We suggest modifications of these criteria using LV mass and volume *Z*-scores. These modified selection criteria will have to be validated in future studies.

## Supplementary Information

Below is the link to the electronic supplementary material.

Supplementary file1 (PDF 407 kb)

## Abbreviations

ASD: Atrial septal defect
ASO: Arterial switch operation
cc-TGA: Congenitally corrected transposition of the great arteries
EF: Ejection fraction
LV: Left ventricle
LVEDV: Left ventricular end-diastolic volume
LVOTO: Left ventricular outflow tract obstruction
MV: Mitral valve
PAB: Pulmonary artery banding
RV: Right ventricle
TV: Tricuspid valve
VSD: Ventricular septal defect
