# Supplementary material for: Anatomic Repair of Congenitally Corrected Transposition: Reappraisal of Eligibility Criteria
Source: Pediatr Cardiol. 2022 Feb 11;43(6):1214–22. doi: 10.1007/s00246-022-02841-z (PMC9294026; doi:10.1007/s00246-022-02841-z)
Supplement: Supplementary file 1 — Supplementary file1 (PDF 407 kb) [file 246_2022_2841_MOESM1_ESM.pdf]

Supplementary Information

Anatomic repair of congenitally corrected transposition: reappraisal of eligibility criteria

Viktoria HM Weixler*,[1], Peter Kramer*,[2], Peter Murin[1], Olga Romanchenko[1], Mi-Young Cho[1], Stanislav Ovroutski[2], Michael Hübler[1], Felix Berger[2], Joachim Photiadis[1]

[1] Department of Congenital Heart Surgery, German Heart Center Berlin, Berlin, Germany

[2] Department of Congenital Heart Disease/ Pediatric Cardiology, German Heart Center Berlin, Berlin, Germany

* These authors contributed equally to this article

Journal of Pediatric Cardiology

Corresponding author:

Peter Kramer, MD

German Heart Center Berlin

Department of Congenital Heart Disease / Pediatric Cardiology

Augustenburger Platz 1, 13353 Berlin, Germany

Office: +49 3045932800, E-mail: kramerpeter@dhzb.de

Supplemental Table 1. Individual evaluation of eligibility criteria for anatomic repair

Age at repair (years)	LV/RV pressure ratio (%)	MVR (grade)	LV EF (%)	LV mass index (g/m²)	LV mass Z-Score	LVEDV index (ml/m²)	LVEDV Z-Score	LV mass/ volume ratio	LV mass/ volume ratio Z-Score	Early repair failure	Mid-term outcome
7.4	99	1	64	49.6	-0.1	39.3	-1.7	1.3	2.4	no	alive
4.3	95	0	58	**38.8**	-1.3	45.2	-1.1	0.9	-0.1	no	alive
0.4	100	0	59	56.0	2.4	43.3	-0.1	1.3	2.5	no	alive
**18.3**	100	0	**52**	45.3	-1.2	36.9	-2.4	1.2	2.2	no	dead
0.5	103	0	60	52.5	0.7	30.0	-2.2	1.8	5.4	no	alive
0.6	100	0	61	**27.6**	-2.1	26.3	-2.3	1.1	1.1	no	alive
1.1	107	0	**53**	47.3	0.8	35.0	-1.3	1.4	1.4	no	dead
8.8	89	**2**	**50**	48.0	-0.4	38.3	-1.9	1.3	2.3	no	alive
0.7	100	0	67	**38.4**	-0.9	31.7	-1.9	1.2	2.1	no	alive
**17.6**	100	0	61	103.0	4.1	55.0	-1.3	1.9	6.1	**yes**	dead
0.5	100	0	62	**43.2**	-0.2	32.4	-1.8	1.3	2.9	no	alive
1.8	100	0	68	45.3	0.0	34.1	-1.7	1.3	2.8	no	alive
14.1	100	0	59	70.3	1.8	44.6	-1.5	1.6	4.4	no	alive
5.2	110	0	68	48.5	0.1	29.5	-2.4	1.6	4.8	no	alive
7.2	103	0	**51**	79.3	2.8	51.0	-0.9	1.6	4.2	no	alive
2.5	98	0	70	59.6	1.4	32.8	-2.1	1.8	5.8	no	alive
0.8	100	0	55	56.5	1.9	46.2	-0.2	1.2	2.2	no	alive
0.6	103	0	64	78.0	5.1	37.1	-1.1	2.1	7.6	no	alive
6.1	97	0	59	69.5	2.5	39.4	-1.5	1.8	5.5	no	alive
2.1	100	0	58	**40.4**	-0.6	26.9	-2.5	1.5	3.9	no	alive
0.9	106	0	60	48.3	0.5	29.7	-2.1	1.6	4.6	no	alive
0.9	102	0	64	**35.8**	-1.3	22.7	-2.9	1.6	4.4	**yes**	alive
0.8	100	0	56	48.5	0.3	46.2	-0.6	1.1	1.2	no	alive
0.9	102	0	55	**44.2**	0.1	28.9	-2.1	1.5	4.1	no	alive
3.2	96	0	63	55.7	0.8	37.9	-1.7	1.5	3.7	no	alive

Values not meeting recommended criteria for anatomic repair of congenitally corrected transposition of the great arteries (age at repair <15 years, systolic LV/RV systolic pressure ratio >70%, mitral valve regurgitation ≤mild, LV EF ≥55%, LV mass index ≥45 g/m² and LV mass/volume ratio >0.9.) are presented in bold [1-6]. Additionally, Z-Scores for LV mass, LVEDV and LV mass/volume ratio are tabulated [7]. Patients who did not meet all of the recommended criteria are shaded. Primary endpoint of early repair failure (given in bold) was defined as early postoperative mortality and/or early postoperative LV dysfunction requiring mechanical circulatory support.
EF=ejection fraction; LV=left ventricle; MVR=mitral valve regurgitation grade 0-3 (0=none/trivial, 1=mild, 2=moderate, 3=severe); RV=right ventricle

References

1. Ibrahimiye AN, Mainwaring RD, Patrick WL, Downey L, Yarlagadda V, Hanley FL. Left Ventricular Retraining and Double Switch in Patients With Congenitally Corrected Transposition of the Great Arteries. World Journal for Pediatric and Congenital Heart Surgery. 2017 Mar;8(2):203–9.

2. Myers PO, del Nido PJ, Geva T, Bautista-Hernandez V, Chen P, Mayer JE, et al. Impact of Age and Duration of Banding on Left Ventricular Preparation Before Anatomic Repair for Congenitally Corrected Transposition of the Great Arteries. The Annals of Thoracic Surgery. 2013 Aug;96(2):603–10.

3. Zartner PA, Schneider MB, Asfour B, Hraška V. Enhanced left ventricular training in corrected transposition of the great arteries by increasing the preload. European Journal of Cardio-Thoracic Surgery. 2016 Jun;49(6):1571–6.

4. Hiramatsu T, Matsumura G, Konuma T, Yamazaki K, Kurosawa H, Imai Y. Long-term prognosis of double-switch operation for congenitally corrected transposition of the great arteries. European Journal of Cardio-Thoracic Surgery. 2012 Dec 1;42(6):1004–8.

5. Imamura M, Drummond-Webb JJ, Murphy DJ, Prieto LR, Latson LA, Flamm SD, et al. Results of the double switch operation in the current era. The Annals of Thoracic Surgery. 2000 Jul;70(1):100–5.

6. Winlaw DS, McGuirk SP, Balmer C, Langley SM, Griselli M, Stümper O, et al. Intention-to-Treat Analysis of Pulmonary Artery Banding in Conditions With a Morphological Right Ventricle in the Systemic Circulation With a View to Anatomic Biventricular Repair. Circulation. 2005 Feb;111(4):405–11.

7. Lopez L, Colan S, Stylianou M, Granger S, Trachtenberg F, Frommelt P, et al. Relationship of Echocardiographic *Z* Scores Adjusted for Body Surface Area to Age, Sex, Race, and Ethnicity: The Pediatric Heart Network Normal Echocardiogram Database. Circulation: Cardiovascular Imaging [Internet]. 2017 Nov [cited 2020 Apr 18];10(11). Available from: https://www.ahajournals.org/doi/10.1161/CIRCIMAGING.117.006979

Supplemental Table 2: Intraoperative and postoperative variables

Variable	Patients N=25)
**Intraoperative characteristics**	
Cardiopulmonary bypass time (min)	286 [236;374]
Cross-clamp time (min)	153 [137;216]
Cardiopulmonary bypass temperature	
Moderate hypothermia (32-28°C)	18 (72%)
Deep hypothermia (<28°C)	7 (28%)
**Postoperative course**	
Intensive care unit stay (days)	7 [5;11]
Duration of chest drainage (days)	12 [7.5;11]
Mechanical ventilation (days)	5 [4;7]
Length of hospital stay (days)	16 [12;21]
**Complications**	
Tachyarrhythmia	6 (24%)
Bradyarrhythmia	4 (16%)
Pacemaker implantation	3 (12%)
Infection	10 (40%)
Acute kidney injury	4 (16%)
Capillary leak syndrome	4 (16%)
Chylothorax	3 (12%)
Bleeding	2 (8%)
Extracorporeal membrane oxygenation	2 (8%)
Ventricular assist device	2 (8%)

Continuous data presented as median [interquartile range]; categorical data are presented as frequency (%)

Supplemental Table 3: Multivariable binary logistic regression

Predictor	B	SE (B)	P-value	OR	95% CI
Age	0.18	0.1	0.2	1.2	0.9-1.6
Prior PAB	16.7	26082.2	0.9	177900 65.4	0-.
LVOTO/PS/PA	-16.2	26082.2	0.9	0	0
Surgical technique[a]	34.4	36885.7	0.9	8.9E+1 4	0-.

Summary of multivariable binary logistic regression analysis to predict early failure of anatomic repair of congenitally corrected transposition of the great arteries. Hosmer Lemeshow goodness of fit test indicated good logistic regression model fit (P=0.96).
Regression coefficient B; ASO=atrial switch operation; CI=confidence interval; LVOTO=Left ventricular outflow tract obstruction; OR= Odds Ratio; PA=Pulmonary atresia; PAB=Pulmonary artery banding; PS=Pulmonary stenosis; SE (B)=standard error of B.
[a] ASO=Arterial switch operation vs. other procedures=ASO+LVOTO-resection, Rastelli or aortic root translocation
